# Expression of Concern on “A Novel Model of Cancer-Induced Peripheral Neuropathy and the Role of TRPA1 in Pain Transduction”

**DOI:** 10.1155/2018/5342101

**Published:** 2018-12-10

**Authors:** Pain Research and Management

*Pain Research and Management* would like to express concern with the article titled “A Novel Model of Cancer-Induced Peripheral Neuropathy and the Role of TRPA1 in Pain Transduction” published in *Pain Research and Management* in December 2017 [1], due to the information that the Faculty Board of the Charité—Universitaetsmedizin Berlin, under the advisement of the responsible ombudsperson, has investigated complaints about this article and has decided to initiate a full investigation. This notice may be updated or replaced based on the outcome of the investigation.

Update, December 2020: The research reported in this article has been subject to legal proceedings at the Verwaltungsgericht Berlin (Berlin Administrative Court). The author did not provide the underlying data to the journal.
